# Acknowledging vulnerability in ethics of palliative care – A feminist ethics approach

**DOI:** 10.1177/09697330211072361

**Published:** 2022-02-27

**Authors:** Sofia Morberg Jämterud

**Affiliations:** Department of Thematic Studies, Technology and Social Change, Linköping University, Linköping, Sweden

**Keywords:** Feminist ethics, theory/philosophical perspectives, palliative care, relational autonomy, vulnerability, nursing

## Abstract

Patients in need of palliative care are often described as vulnerable. Being vulnerable can sometimes be interpreted as the opposite of being autonomous, if an autonomous person is seen as an independent, self-sufficient person who forms decisions independently of others. Such a dichotomous view can create a situation where one has experiences of vulnerability that cannot be reconciled with the central ethical principle of autonomy. The article presents a feminist ethical perspective on the conceptualisation of vulnerability in the context of palliative care. It does so through the lens of the concepts of inherent and pathogenic vulnerability from the taxonomy on vulnerability suggested by Mackenzie et al. To differentiate between forms of vulnerability, is important since even though vulnerability can be regarded as a shared life condition it can be the product of practices creating harm to the patient. The article also presents an analysis of how vulnerability can be included in the interpretation of the ethical principle of autonomy, in order to be relevant in palliative care where vulnerability is salient, namely, as relational autonomy. Furthermore, two practical implications for nursing practice are suggested. Firstly, to acknowledge vulnerability as a shared life condition one needs training in order to neither be overwhelmed by one’s own vulnerability, nor become invulnerable when facing vulnerability in others. Secondly, to foster relational autonomy includes navigating between the patient exercising their autonomy within a framework of relations, and shielding the patient from paternalistic practices. Nurses could be particularly suited for this role, which includes creating an environment which is open and supportive; navigating between patient, family and staff; seeing and acknowledging the complex situation in which patient autonomy is actually played out; and promoting patient autonomy.

## Introduction

Cicerly Saunders once stated that she wished for St Christopher’s hospice to be a community where all, patient as well as staff, shared vulnerability.^1^ Since all humans are vulnerable to the uncertainties and fragilities of human life, vulnerability can be seen as a shared life condition. To be vulnerable means that as humans we can be susceptible to harm and injury and that we can be at risk of being wounded, both physically and emotionally. Vulnerability relates to humans as social beings, vulnerable to others’ actions towards us, at some stages more dependent on care, and in certain stages of life at greater risk of being hurt.^2,3^

Palliative care is still associated with end-of-life care.^4^ However, it is broader in scope and is integrated into earlier stages of care of the patient.^5,6^ The focus of palliative care is on holistic care and the particular problems facing patients living with life-threatening illness.^7^ This concerns not only end of life but a longer period of time. Patients who are in need of palliative care, and also their families, are often described as being vulnerable and sometimes even referred to as ‘the most vulnerable’.^8^ However, even though vulnerability is a recognised experience, it has hardly been examined within health care, either as an academic concern, or in regard to its clinical implications.^9^ Furthermore, in the field of palliative care ethics, it is very rare encountering discussions on the conceptualisation of vulnerability and how vulnerability can have a bearing on the understanding of central ethical principles. However, for a practice such as palliative care – where vulnerability is salient – such an examination is of importance.

To be vulnerable can sometimes be interpreted as the opposite of being autonomous if the autonomous agent is seen as an independent and self-sufficient person who forms his or her decisions independently of others.^10^ Such a view would indicate that the more vulnerable you are, the less autonomous you are. Such a dichotomous view can create problems for practices like palliative care where patients are recognised as vulnerable. On the one hand, many patients in palliative care experience vulnerability in the form of dependence and reliance on others, but on the other hand, it is of the utmost importance to uphold respect for the patients’ autonomy. A dichotomous view of autonomy and vulnerability raises important questions both for palliative care ethics and clinical practice such as: what can vulnerability mean in the context of palliative care and how can vulnerability be reconciled with the principle of autonomy?

The first aim of this article is to present an analysis of two different forms of vulnerability in the context of palliative care. To differentiate between forms of vulnerability is important since even though vulnerability in one sense can be regarded as a shared life condition and thus unavoidable for all humans, vulnerability is in another sense the product of practices creating harm to the patient and ought to be avoided. The second aim is to present how vulnerability can be included in the interpretation of the ethical principle of autonomy, in order to be relevant in palliative care where vulnerability is salient. This could be done when the ethical principle of autonomy is interpreted as relational autonomy. Human beings are inherently vulnerable, and from this follows an understanding that human beings are also dependent on and situated in relation to others. Moreover, in adapting a relational concept of autonomy, vulnerability and autonomy do not stand in opposition to each other.

### Background

In recent decades, the concept of vulnerability and its normative dimensions have gained recognition within fields such as bioethics,^2,11,12^ feminist ethics,^3,13-15^ and nursing ethics.^16,17^ Within the field of bioethics, vulnerability has been a central discussion point in questions about research ethics and has primarily concerned the identification of vulnerable groups or populations that are in need of extra protection in order not to be exploited. Vulnerable groups are often referred to as those not able to provide informed consent and not having the ability to protect their own interests.^12^ This can be noticed in UNESCO’s *Universal Declaration on Bioethics and Human Rights* (2005)^18^ where respect for vulnerability is underlined in relation to applying medical practice and where it is stated that ‘Individuals and groups of special vulnerability should be protected’. However, criticism has been voiced concerning the overhanging risk of stereotyping certain groups and populations when labelling them as vulnerable.^19^ Regarding research on vulnerability in palliative care ethics, important research has been done from the perspective of vulnerable groups and the socio-ethical problem of vulnerability as a product of injustice. This research has shown that marginalised groups, groups with structural vulnerability, have unequal access to palliative care.^20-22^ Such research also calls for strategies and policies in order to ensure the equal right to palliative care.^20^ Furthermore, important work has been done on how palliative care teams can organise care in order to offer inclusive care and enable access to palliative care for vulnerable groups.^23^

Scholars within feminist ethics and nursing ethics have broadened the perspective on vulnerability from only being a characteristic of certain groups and populations to acknowledging vulnerability as an inherent condition of, not just some, but all human beings.^24^ Vulnerability therefore could be understood as a life condition. Interpreted thus it is connected to embodiment and the fragility linked to being a biological being and hence vulnerable, for example to illness and death.^25^ Ethicists have also pointed to the fact that to be vulnerable underlines the possibility of being hurt or wounded by someone or something, but at the same time, there is also a possibility of preventing harm or protecting someone from harm. Accordingly, vulnerability can be understood as a relational concept and it is claimed that due to this relationality the normative dimensions of vulnerability could be explored.^2,26^

However, the understanding of vulnerability as a life condition and how a recognition of this life condition could impact ethics of palliative care is important to examine within a form of care where vulnerability is salient.

## Inherent and pathogenic vulnerability and the clinical context of palliative care

The feminist ethicists Catriona Mackenzie, Susan Dodds and Wendy Rogers have together developed a theoretical framework for vulnerability, a taxonomy, and have extensively discussed the role of vulnerability within bioethics.^3,12^ The taxonomy consists of three different sources of vulnerability: inherent, situational and pathogenic.^3^

This taxonomy is of value since it deals with aspects of vulnerability that are ethically challenging and it addresses vulnerability in relation to autonomy, which is of particular interest in this article. In relation to the aim of the article, only inherent and pathogenic vulnerability will be examined as the first notion captures vulnerability as a life condition, which will be discussed in relation to autonomy, and the notion of pathogenic vulnerability helps examine coercive practices which can be an overhanging risk in acknowledging vulnerability. In the following, inherent and pathogenic vulnerability will be applied to the context of palliative care.

### Inherent vulnerability

Mackenzie et al. state that: Inherent vulnerability refers to sources of vulnerability that are inherent to the human condition (p. 7).^3^ These vulnerabilities arise due to human life being conditioned by corporality resulting in our dependence on others as well as our dependence on material needs being met. To be a corporeal being is to be vulnerable to decay, decline and illness. Inherent vulnerability therefore pinpoints the fragility facing all human life.^3^ In relation to how inherent vulnerability can be understood and contextualised in palliative care, corporality forms an important focus. During various stages of a patient’s course of disease, the situation when living with life-limiting illness is characterised by weak physical condition and bodily decay, especially during the dying phase. A patient needs to face bodily changes, in a brutal way sometimes. Different forms of affliction can remind us of our limitations regarding control of our bodies, and illness can starkly confront us with our inherent vulnerability. A plethora of illness narratives describing difficulties with life limiting illness have been written. In one of these, the author describes several changes that make him scared: bodily decay, losing bodily functions, changed appearance, and losing control.^27^ So, understanding inherent vulnerability in palliative care is to understand the patient’s situation when facing bodily changes, but also to recognise that this situation can create a real threat to the patient’s view of themselves. The experience of being estranged to one’s body can have a very negative impact on one’s feeling of self.^28^ Research has also shown that for many patients in palliative care the deterioration process alters their feeling of identity, and can have such an impact that there develops a stark rift between the feeling of self before and after one’s illness.^29^

As noted earlier, the taxonomy links dependency and inherent vulnerability and underlines that vulnerability is a common human life condition since dependence is an inevitable condition shared by all humans and stresses that we are relational beings.^3^ However, even though dependence is a common characterisation of human life, and not only a negative characterisation, within palliative care certain forms of dependence can be very problematic. Dependence on others as a result of one’s physical inability can be closely related to the experience of oneself as a burden to, for example, relatives and the severe and troubling feeling of losing one’s independence.^29^ This aligns with Mackenzie et al. who underline that inherent vulnerability can vary in a person’s life in relation to different factors such as disability and health, and also depends on factors such as resilience, capacity to cope and whether the person has a network of social support.^3^

Inherent vulnerability underlines vulnerability as a common life condition and highlights that we are all vulnerable to illness and ultimately death and also recognises our dependence on others – for good or bad.

### Pathogenic vulnerability

Another source of vulnerability in the taxonomy is described as pathogenic and this is generated by harmful responses to vulnerability which in turn can intensify vulnerability. Rogers et al. exemplify this in the following way:

There are a variety of sources of pathogenic vulnerability. Pathogenic vulnerability may be generated by morally dysfunctional interpersonal and social relationships characterized by disrespect, prejudice, or abuse, or by socio-political situations characterized by oppression, domination, repression, injustice, persecution, or political violence (p. 25).^12^

Pathogenic vulnerability is of the utmost importance to discuss in relation to palliative care since it concerns responses to vulnerability, both interpersonal and socio-political. As mentioned earlier, within palliative care, all patients are dependent on others and in need of care and support because of one’s physical condition, even though this can vary in degree. Due to severe illness, patients can be less able to protect themselves from abuse in different forms. Research points to patients living with life-limiting illness experience loss of self-respect, dignity, integrity and sense of self when others treat them in disrespectful ways. Patients could experience a transformation from being regarded as a subject and worth listening to – before being ill – to experiencing an objectification, when living with life-limiting illness.^30^

Pathogenic vulnerability can take many forms, including paternalism. If vulnerability is linked with helplessness and victimhood, there is an attendant risk that paternalistic and coercive forms of interventions may be regarded as acceptable in the protection of vulnerable persons.^3,31^ There are many definitions of paternalism but as a general idea medical paternalism can be understood as a decision being made by health care professionals instead of the patient, with the aim of promoting the good. In situations where patients can have decreased capacity for decision-making due to their serious physical condition, paternalism can be understood in a weaker form.^32^ It is also important to point to those situations where the patient is less regarded or recognised as autonomous due to bodily deterioration and therefore has more difficulty being taken seriously and listened to, a devasting consequence for the patient. Mackenzie et al. point to that such a risk can be greater in relations of inequality.^3^ This situation has also been shown in empirical studies in recent years. In one of these studies, staff in palliative teams were interviewed about dignity and autonomy, and it was shown that the patient’s severe illness could have an effect on the respect for the patient’s autonomous choice:

[…] and sometimes it seems like at the same time as one’s autonomy is degraded, the possibility to take care of oneself is degrading, then it is like… it is more difficult to be regarded as being autonomous, to have the right to express one’s autonomous choices and decisions and to have others really respect this. It sometimes seems like the one who slurs or has other physical difficulties, has more difficulty with being taken seriously (p. 101).^33^

Vulnerability has sometimes been regarded as something to overcome or reduce but the intrinsic form points to vulnerability as being part of the human condition, a life condition, and not a state to overcome or abolish. However, even though vulnerability in one sense is unavoidable in a palliative care context due to, for example, the physical condition of the patient, vulnerability in another sense can be the product of practices creating harm to the patient. Of particular importance is the risk that a person who is regarded as vulnerable also has problems being regarded as autonomous. There is then a risk of pathogenic vulnerability. It is therefore of importance in palliative care ethics to formulate a view of autonomy that is in congruence with the view of the vulnerable self in order to avoid harm to the patient.

## Inherent vulnerability and relational autonomy in palliative care

As has been noted, patients in palliative care are sometimes regarded as the most vulnerable^8^ and as seen different forms of vulnerability come to the fore in palliative care. Furthermore, a starting point in this article is the understanding that humans are inherently vulnerable, and particularly important aspects of this are embodiment and the self as situated in relation to others and dependent on others. Any interpretation of autonomy depends on one's view of the human being^34^ and if adhering to a view of the self as inherently vulnerable this has implications for the understanding of autonomy and autonomous decision-making. As noted earlier, vulnerability and autonomy are sometimes understood as standing in opposition to each other, creating a problem of how to reconcile the two. However, as has been claimed by many feminist ethicists, the view of the vulnerable self could stand in congruence with relational approaches to autonomy,^35^ which this article claims is of importance for palliative care ethics.

Relational approaches to autonomy do not reject the idea of autonomy as such but can embrace an idea that “to be autonomous is to be able to make choices and act in line with one’s reflectively endorsed beliefs, values, goals, wants, and self-identify” (p. 43).^10^ Even though relational approaches to autonomy represent a heterogeneous field,^36^ certain common characteristics can be notified. Firstly, the common criticism from relational approaches to autonomy is that traditional views on autonomy have taken an excessively individualistic view of the self.^34,10^ Instead, these approaches would highlight that individuals are socially situated and that it is important to examine social and cultural dimensions and their relation to autonomy as well as to acknowledge intersubjective aspects.^35,36^ Since relational approaches suggest that the self is relational this means that if and when autonomy is described as ‘self-determination’ the self that is described is relational as well as social, and that choices, beliefs, values and goals are always developed and enacted in a social and cultural context.^10^ Hence, it is not autonomy as such that is regarded as problematic but certain interpretations of the view of the autonomous self and the view of autonomous decision-making.

If the autonomous self is mainly understood in terms of self-sufficiency and independence, then such a view can be understood as standing in contrast to the vulnerable self. The autonomous self is, so to speak, invulnerable^13^ since the view of the vulnerable self stresses that the self is situated in relation to others and dependent on others. Feminist ethicists have pointed to the dichotomy between autonomy and vulnerability.^3^ The view of the vulnerable self also has implications for interpretations of the meaning of autonomous decision-making. Relational approaches to autonomy forms critique against a view of the decision-making process where an independent, self-sufficient person reaches a decision without interactions from others in forming their decisions, and it is sometimes even said the patient ought to be shielded from interference or external coercion.^34,37^ Research has pointed out that this view of an autonomous decision-making process is not consistent with the relational character of much healthcare,^38^ such as in palliative care.^39,40^ Empirical studies with patients in the end of life has also pointed to the importance of relations in a decision-making process showing that shared decision-making was of significant importance to both patients and relatives.^41^

The perspective of relational autonomy points, as seen, to the importance of decisions being enacted in relation to others and being included in a social context. Conceptions of relational autonomy show that developing and exercising autonomy is not detached from our social relationships; on the contrary, social relationships can shape decisions and choices that are made by the patient, sometimes in a responsible way but sometimes they can also constrain these choices and sometimes the influence of others can be harmful to the patient’s autonomy.^35^ Social relationships cannot be simplified as either enhancing or limiting autonomous decision-making but they are complex. Relational autonomy therefore underlines that since the social context and social relations can play a role in autonomous decision-making it is important to analyse these relationships and how they contribute to the process of decision-making. To regard autonomous decision-making from the perspective that an individual is embedded in social relations, for good or bad, differs from an individualistic perspective where non-interference from external agents is regarded as an important aspect of autonomous decision-making.^42^ A relational autonomous approach can help pinpoint the risk of oppression of the patient’s autonomy, which can lead to a pathogenic vulnerable situation. If relational aspects of autonomy are not regarded the risk can be that one loose tools for analysing oppression to the patient, adding to the pathogenic vulnerability of a patient.^43^

In conclusion, the following gains and preventable harms that can occur by recognising vulnerability in palliative care can be mentioned: By analytically addressing different forms of vulnerability, as in this article from the taxonomy by Mackenzie et al., a language is suggested which makes it possible to identify vulnerability within palliative care. This is vital since vulnerability is both a shared life condition (inherent) *and* a product of practices creating harm to the patient (pathogenic) and therefore demands a variety of different moral responses. Furthermore, to acknowledge vulnerability can improve and give alternative views on the ethics of palliative care, especially in relation to the central ethical principle of autonomy. In the form of relational autonomy, the patient as *both* inherently vulnerable and autonomous is recognised, and since vulnerability and autonomy do not stand in opposition to each other but can be reconciled, this form of autonomy is relevant for palliative care. Relational autonomy reconciles the patient’s situation of being dependent on care, receiving help while at the same time having one’s autonomy respected. Furthermore, it recognises that autonomy is played out in relations.

## Clinical implications

Palliative care has always included an existential perspective, that it is vital to also care for the existential needs of the patients. To recognise one’s own vulnerability as a human being, that human life is vulnerable to death, illness, suffering, can be claimed to be a shared existential experience of both patients and staff in palliative care since the finitude and fragility of life are clear in this form of care. However, it has been noted that the lives of health care professionals, as they are exposed to and witness suffering and illness, can be deeply impacted by patients’ vulnerability and also impacted by the limits one has in treating or reducing suffering.^44^ To acknowledge vulnerability could be seen as a way to continue to shape palliative care practice as a form of care where the existential needs of the patient are addressed. However, to be able to do that it is important to attend to the caregivers’ own existential concerns. In a recent European Association for Palliative Care white paper the importance of developing a reflective capacity about one’s own existential concerns was raised and the authors point out: “Self-awareness can help the healthcare practitioner to avoid being distracted by their own fears, prejudices and restraints and attend to the patient”.^45^ This underlines the importance of finding ways to acknowledge one’s own vulnerability as a human being in order to be able to provide good care. Hence, neither be overwhelmed by one’s vulnerability nor become invulnerable and block one’s vulnerability facing serious illness and death. Instead find ways to recognise vulnerability also as a professional.^44^ Vulnerability is sometimes regarded as central in nursing both in the sense of preventing patients from harm but also in the sense of facing one’s own vulnerability in order not to dehumanise others.^46^ Carel claims that: “… vulnerability may require more recognition by the profession. Working as a nurse brings with it an almost daily reminder of the fallibility of human flesh and sprit and the fragility of human life and goods. This, in turn, is a lesson in vulnerability” (p. 218).^47^ To develop a reflective awareness needs training, both on an individual level as a nurse but also on a collegial level since meeting the existential needs of patients is a central part of a team-based palliative care practice.

*The ICN code of ethics for nurses* emphasize education on ethical principles such as the principle of autonomy.^48^ It has been suggested that nurses ought to develop “…. a more holistic understanding of autonomy, which supports patient agency and identity in everyday care” (p.1024).^39^ In this article, it has been claimed that inherent vulnerability, which underlines the self as situated in relation to others and dependent on others, can be combined with respect for autonomy in the form of relational autonomy. However, this approach is demanding and can be challenging in clinical practice since it requires navigating between situations where the patient is exercising autonomy within a framework of responsible relations *or* shielding the patient from pressure, abuse or paternalistic practices from family members or health care personnel, hence pathogenic vulnerability.^35^ It can also be important in clinical practice to be observant regarding when the patients’ own experience of their vulnerability creates a situation where they regress and refrain from exercising their autonomy. To foster relational autonomy therefore needs a trusting interpersonal environment, and continuous interpersonal discussions both within the team as well as with family and patients. Nurses could be particularly suited for such a role which includes creating an environment which is open and supportive; navigating between patient, family and staff; seeing and acknowledging the complex situation that patient autonomy actually is played out in; and promoting patient autonomy. Nurses are well suited for this role, also within interprofessional teams, since they often have established relations with patients and family, follow the patients’ daily life closely, and also are trained to enhance cooperation and negotiation.^49^ Concrete instruments that can promote relational autonomy have been suggested whereof advanced care planning is one. As noticed by Killackey et al. this planning can be done in such a way that it both recognises vulnerability and promotes the patient’s autonomy as played out in relations.^50^
